# Pediatric Respiratory Pathogens Circulate in Children and Adults in Communities Near Susceptible Wild Great Ape Populations in Uganda

**DOI:** 10.1155/2024/1771163

**Published:** 2024-10-23

**Authors:** Patrick Tusiime, Taylor Weary, Tressa Pappas, Shamilah Tuhaise, John Walter Akankwasa, Daniel Sempebwa, Emily Otali, Caroline Asiimwe, Matthew R. McLennan, Gladys Kalema-Zikusoka, Elizabeth Ross, James Gern, Tony Goldberg

**Affiliations:** ^1^The Kasiisi Project, Fort Portal, Uganda; ^2^Department of Pathobiological Sciences, University of Wisconsin–Madison, Madison, Wisconsin, USA; ^3^Department of Pediatrics, University of Wisconsin–Madison, Madison, Wisconsin, USA; ^4^Budongo Conservation Field Station, Masindi, Uganda; ^5^Kibale Chimpanzee Project, Fort Portal, Uganda; ^6^Jane Goodall Institute Uganda, Kampala, Uganda; ^7^Bulindi Chimpanzee and Community Project, Bulindi Town Council, Bulindi Town, Uganda; ^8^School of Law and Social Sciences, Oxford Brookes University, Oxford, UK; ^9^Conservation Through Public Health, Entebbe, Uganda

## Abstract

Respiratory infections are a leading cause of death in developing countries. Infants and young children are especially susceptible to disease because they lack immunity, whereas adults who have acquired immunity can be infected asymptomatically. Great ape species, all of which are endangered, are similarly susceptible to respiratory illnesses caused by human respiratory pathogens. We obtained 432 nasopharyngeal swab samples (127 from adults and 305 from children) in a cross-sectional study that took place between February and October 2022 at four sites in Western Uganda (Budongo Central Forest Reserve, Bulindi Town Council, Bwindi Impenetrable National Park, and Kibale National Park) where the participants live in communities where interaction with apes is frequent. Prior research at Kibale has shown that locally circulating human respiratory pathogens have led to multiple lethal outbreaks in wild eastern chimpanzees (*Pan troglodytes schweinfurthii*). We used a multiplex PCR panel to characterize respiratory pathogens, with the goal of assessing whether respiratory illnesses in the chimpanzees of Budongo and Bulindi and the mountain gorillas (*Gorilla beringei beringei*) of Bwindi might have originated in local children and been introduced to the apes via asymptomatic adult carriers. The prevalence of respiratory pathogens was twice as high in Bwindi (44.0%) as it was in Budongo (24.0%) and Bulindi (20.8%), while the prevalence was intermediate at Kibale (34.4%). Rates of respiratory pathogen detection were higher but statistically indistinguishable in children compared to adults at Budongo and Bulindi, and children were 15.0 times more likely than adults to have positive detections at Kibale. At Bwindi, however, the pattern was reversed, with adults 2.6 times more likely than children to have positive detections. Rhinovirus, metapneumovirus, human parainfluenza virus 3, respiratory syncytial virus, and coronavirus OC43, all of which have been identified as causative agents of respiratory disease outbreaks in great ape populations across sub-Saharan Africa, accounted for three quarters (73.6%) of detected pathogens. Our data support the idea that human respiratory pathogens that can infect great apes occur at high frequencies in human populations in Western Uganda that live close to and interact with wild apes that have suffered from lethal outbreaks caused by these same pathogens. Reducing respiratory infections in local children, thereby reducing both carriage of those infections into the forest by people and ape exposure to these pathogens when they enter human spaces, should decrease the risk of respiratory disease outbreaks in apes.

## 1. Introduction

The global burden of respiratory pathogens in children is a significant public health concern, leading to substantial morbidity and mortality in low- and middle-income countries (LMICs) [1], particularly in children under the age of five [2, 3]. Viruses that circulate regularly among young children and cause upper respiratory tract infections (URIs), collectively referred to as the “common cold,” are responsible for a significant portion of this burden [4, 5]. Most studies have examined patterns of respiratory pathogens in children in high-income countries with temperate climates [6, 7]. However, little is known about the epidemiological and clinical features of respiratory infections in children in LMICs, even though acute respiratory infections have a significant impact on child mortality in these countries, where 90% of deaths caused by URIs occur [8]. Data from sub-Saharan Africa are particularly scarce [9].

Great apes in the wild are also susceptible to outbreaks of human respiratory pathogens that plague children worldwide [10–18]. Clinical signs of affected animals can be severe and even lead to death [18–22]. Across wild ape field research and tourism sites, respiratory illnesses are among the primary known causes of death [13, 21, 23, 24]. Great apes are especially susceptible to human respiratory diseases because of their close genetic and physiological similarity to humans, and concern over these outbreaks is growing due to their ability to diminish or even extirpate already small and fragmented wild populations [10, 11, 25]. Currently, very little is known about how common these viruses are in human communities in many areas of sub-Saharan Africa where humans and great apes interact in ways that could facilitate reverse zoonotic respiratory disease transmission, although efforts to evaluate this important human–animal interface are increasing [26, 27].

Recently, we reported data on the frequency of respiratory symptoms [28] and viral infections [29] in rural Western Uganda near Kibale National Park, in which the largest single population of Uganda's chimpanzees reside [18, 30–32]. For these previous studies, monthly data on respiratory symptoms and nasopharyngeal swabs were collected from adults working in Kibale and young children from villages within 5 km of the park from May 2019 to July 2022. The incidence rates of respiratory infections in children were twice as high as the global average for 2019, but comparable for adults. Younger children had the highest frequencies and severity of respiratory infections compared to older children or adults. Respiratory illnesses peaked at the beginning of each school trimester, coinciding with children returning to classrooms after a 1–2 months-long holiday. Interestingly, the incidence of respiratory infections in children decreased significantly during the COVID-19 lockdown, indicating the impact of public health measures on disease incidence. Rhinovirus (RV), metapneumovirus (MPV), and parainfluenza virus 3 (PIV 3), the causative agents of lethal chimpanzee respiratory disease outbreaks in Kibale in 2013 [18] and 2017 [15], were detected in children and adult forest workers. Rhinovirus was the most prevalent pathogen detected throughout the study, consistent with previous studies worldwide.

**Figure 1:**
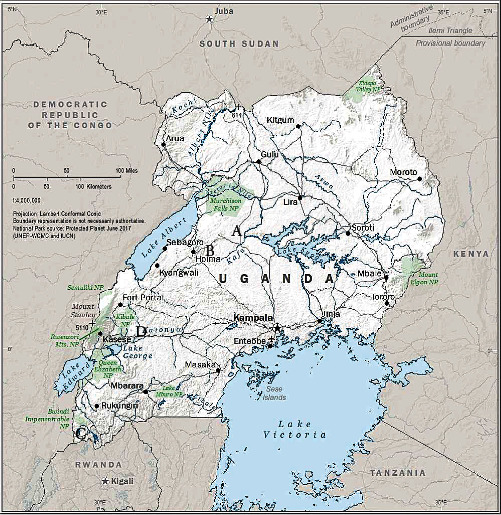
Map of Uganda indicating locations of communities where adults and children were sampled, all of which are located near wild great ape habitats (A: Budongo Central Forest Reserve; B: Bulindi Town Council; C: Bwindi Impenetrable National Park; and D: Kibale National Park).

Here, we investigated the generalizability of our previously published findings by characterizing respiratory pathogens in children and adults from other Ugandan communities near wild great ape populations. Our goal was to examine whether the epidemiological transmission pathway we identified previously (pathogen circulation in local children leading to infection of wild apes through the carriage of pathogens into the forest by asymptomatically infected adults) was occurring widely across western Uganda. We describe patterns of respiratory pathogen infections in human communities near great ape habitats in Uganda: Budongo Central Forest Reserve, Bulindi Town Council, Bwindi Impenetrable National Park, and Kibale National Park (Figure 1). While Budongo, Bwindi, and Kibale are large, protected forests bordered by human communities, Bulindi is an unprotected landscape where chimpanzees use remnant forest patches within the confines of villages [33]. Apes leave the protected areas periodically to forage in crop fields and villages [34–36], but at Bulindi, the chimpanzees live wholly within the confines of villages in close proximity to people, including symptomatic children and asymptomatic adults, thereby increasing their risk of reverse zoonotic transmission. We hypothesized that patterns of infection related to age and symptomology would be similar among all four sites, which span Ugandan's western border and represent the eastern edge of Africa's great ape-containing forests [37–40]. We also sought to compare our results to those from other communities around the world, not only to place human respiratory infections in Uganda in a broader global context but also to identify commonalities to the risk of reverse zoonoses to apes elsewhere in Africa and around the world [41]. Our ultimate goal is to provide evidence for designing interventions to improve human health and to protect vulnerable great ape populations living nearby. By doing so, we employ the One Health approach, which acknowledges and capitalizes on the fact that human, animal, and environmental health are interrelated [42–44], to advance crucial public health and conservation initiatives.

## 2. Materials and Methods

### 2.1. Ethical Approval

Samples were collected under the guidelines of the World Medical Association Declaration of Helsinki and shipped with institutional approval from Makerere University, School of Health Sciences (2018-077) and the Uganda National Council for Science and Technology (NS 657). With institutional approval from the University of Wisconsin–Madison (2019-0229-CR003), deidentified data were analyzed. Every participant gave voluntary consent to participate. Adult participants and the parents of participants who were children (those under the age of 18 years) gave parental consent. Children who were 8 years old or older gave assent. Native speakers conducted all conversations about informed consent/assent in Runyoro, Rutooro, or Rukiga, the native languages of the study participants.

### 2.2. Study Sites, Subjects, and Sample Collection

We enrolled children (*n* = 269) and an adult comparison group (*n* = 127) who reside and work in or close to great ape habitats in Budongo Central Forest Reserve, Bulindi Town Council, Kibale National Park, and Bwindi Impenetrable National Park in Western Uganda (Figure 1). These locations are well-known for being conservation priority locations for gorillas (Bwindi) and chimpanzees (Kibale and Budongo) as well as a comparative site where wild chimpanzees live and forage entirely in and around human villages (Bulindi). Bwindi, Kibale, and Budongo sites are also popular ecotourism destinations not only due to the presence of apes but also because of their natural beauty and high biodiversity [25]. Activities related to apes offer major employment opportunities in the nearby communities and therefore contribute to the local economies [45]. As human populations expand, proximity, interactions, and conflict between people and apes are also increasing [46, 47]. One consequence of these interactions is an increasing frequency of respiratory disease in the ape populations, caused by pathogens transmitted from humans to apes [25].

Upon receiving parental and informed consent and obtaining the necessary information, local medical professionals proceeded to gather nasopharyngeal swabs. These swabs were then placed in RNAlater preservation solution (Thermo Fisher, Waltham, MA, USA) and stored in −20°C freezers before being shipped for analysis using the Luminex NxTAG Respiratory Pathogen Panel (Luminex Corporation, Austin, TX, USA), a multiplex PCR assay targeting 20 of the most common human respiratory pathogens, including influenza viruses A and B, rhinovirus/enterovirus, adenovirus, parainfluenza viruses 1–4, coronaviruses (CoV NL63, CoV 229E, CoV HKU1, CoV OC43, and SARS-CoV-2), respiratory syncytial viruses A and B, metapneumovirus, human bocavirus, and the bacterial pathogens *Chlamydophila pneumoniae*, *Mycoplasma pneumoniae*, and *Legionella pneumophilia*. Sensitivity and specificity vary by pathogen but on average are approximately 95% and 99%, respectively [48]. Rhinoviruses were typed by partial sequencing as described elsewhere [48].

At the time of swab collection, each participant from Budongo, Bulindi, and Kibale was asked if they were currently experiencing or had any respiratory symptoms in the past 2 weeks using a survey instrument described previously [28]. Queried symptoms included cough, runny nose, sneezing, sore throat, fever, wheezing, and dyspnea. Study personnel conducted these interviews in the participants' local language, either Rutooro or Runyoro. Symptoms data were not available from participants at Bwindi due to site-specific constraints.

### 2.3. Inferential Statistics and Software

Parametric model assumptions were assessed with Shapiro–Wilk tests for verification of normality and with Levene's test for verification of homogeneity of variances in R [49]. Symptoms status, respiratory pathogen infection, age class, and cohort were compared by *Chi*-squared test or Fisher's exact test for association. A two-sided *p*-value of less than 0.05 was regarded as statistically significant. The map of study sites was generated using a public domain map of Uganda from the United States National Geospatial-Intelligence Agency (https://www.nga.mil) [50].

## 3. Results

In total, 432 nasopharyngeal swabs (127 from adults and 305 from children) were collected from the four study sites (Figure 1) between February and October 2022 (Table 1). Presence or absence of respiratory symptoms was recorded for each participant at Budongo, Bulindi, and Kibale, but not Bwindi, at the time of swab collection (see Section 2). The most frequent presentation was a nonviral cold (S+/P−), defined as the presence of respiratory symptoms but no respiratory pathogen detected (58.9% in adults and 59.4% in children; adults vs., children n.s.) (Table 2). Children were more likely to have symptomatic respiratory infections (S+/P+) than adults (26.5% vs., 11.0%; *χ*^2^ = 6.9, df = 1, *p*=0.0088), while adults were more likely to have neither respiratory symptoms nor detected pathogens (S−/P−) (28.8% vs., 11.0%; *χ*^2^ = 14.7, *df* = 1, *p*=0.0001) (Table 2). Respiratory pathogen prevalence was roughly twice as high at Bwindi (44.0% positive detections) compared to Budongo (24.0%) and Bulindi (20.8%) (*χ*^2^ = 15.9, *df* = 3, *p*=0.0012), while prevalence at Kibale was intermediate (34.4%). Rates of respiratory pathogen detection were lower but statistically indistinguishable in adults compared to children at Budongo (Table S1; OR with 95% CI = 0.4 [0.1, 1.4], *p*=0.1776) and Bulindi (Table S2; OR with 95% CI = 0.7 [0.2, 2.2], *p*=0.6020). At Kibale, children were 15.0 times more likely to have positive detections than adults (Table S4; OR with 95% CI = 15.0 [2.2, 644.3], *p*=0.0006). At Bwindi, however, adults were 2.6 times more likely to have positive detections than children (Table S3; OR with 95% CI = 2.6 [1.1, 6.5], *p*=0.0265).

**Figure 2:**
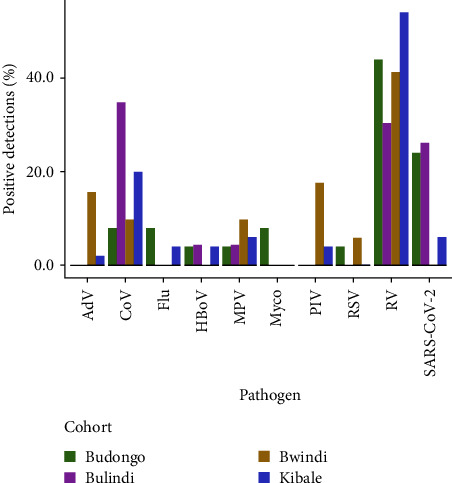
Prevalence of respiratory pathogens among swabs with positive detections at each of the four sites. AdV—adenovirus; CoV 229E, CoV NL63, CoV HKU1, and CoV OC43—“common cold” coronavirus; Flu—influenza virus; HBoV—human bocavirus; MPV—metapneumovirus; Myco—*M. pneumoniae*; PIV 1, PIV 3, and PIV 4—parainfluenza virus; RSV—respiratory syncytial virus; RV—rhinovirus; SARS-CoV-2—severe acute respiratory syndrome coronavirus 2.

Across all study sites, 15/20 (75.0%) pathogens on the Luminex Respiratory Pathogen Panel were detected, with only coronavirus 229E, parainfluenza virus 2 (PIV 2), RSV B, and the bacteria *C. pneumoniae* and *Legionella pneumophila* not detected (Tables S1–S4). Rhinovirus (RV) was the most commonly detected virus across sites (50.4% of all detections), except at Bulindi, where coronaviruses, including seasonal alphacoronaviruses and SARS-CoV-2, occurred at the highest prevalence. SARS-CoV-2 was detected at each site except Bwindi (prevalence at Budongo: 24.0%, Bulindi: 26.1%, Bwindi: 0.0%, Kibale: 6.0%). Respiratory pathogens that have been documented as causative agents of respiratory disease outbreaks in African great ape populations (RV, MPV, PIV 3, RSV, CoV OC43) were detected at each site (Figure 2).

## 4. Discussion

Among the virus-positive samples across all study sites, we identified 15 respiratory pathogens ranging in prevalence from 0.7% (influenza A, PIV 1, PIV 4) to 50.4% (RV). This list included 15 out of 20 (75.0%) of the pathogens found on the Luminex Respiratory Pathogen Panel, which is designed to detect the world's most common human respiratory pathogens. Our data from Uganda resemble those from studies performed elsewhere in the world that have detected RV as the causative agent of half of common cold illnesses, both in temperate regions, such as the United States [7] and Finland [6] as well as in subtropical and tropical countries, such as Mozambique [51] and Uganda [29]. *M. pneumoniae* detections were far lower in our data than have been reported in other studies, where positive detections have ranged from 4% to 39% [52]. *M. pneumoniae* infections tend to spike in 3- to 7-year cycles with background endemic circulation in the intervening years [53]. Therefore, it is possible that our study coincided with a period of low *M. pneumoniae* activity in Western Uganda. There were 15 detections (12.8%) of SARS-CoV-2 across all study sites. This relatively high rate of detection may reflect the rapid movement of the Omicron variant into and within Uganda in 2022, even among vaccinated individuals [54].

Over half of all respiratory illnesses (59.3%) were pathogen negative (S+/P−), a rate higher than we previously observed at Kibale (50.3%) [29], and over twice as high as rates in high-income countries with temperate climates, such as the United States (25.3% [55]; 22.0% [56]) or Finland (31.0%) [57]. These nonviral colds may be a consequence of high concentrations of both indoor (i.e., cookstove smoke) [58] and outdoor (i.e., dust, vehicle exhaust, smoke) air pollution [59] in rural Uganda, even in areas adjacent to national parks, which can exacerbate asthma in children [60] and chronic obstructive pulmonary disorder (COPD) in adults [61]. Regardless of etiology, the high prevalence of nonviral colds means that symptoms-based screening for forest workers entering ape habitats [62] is an imprecise way to prevent apes from becoming exposed to human pathogens. Symptomatic forest workers may not be shedding respiratory pathogens, but, critically, asymptomatic infections (S−/P+), during which forest workers could be shedding meaningful amounts of virus [63], are going undetected. Because routine molecular diagnostic testing for these pathogens is not currently feasible at most field sites, we support recent calls to ensure forest workers follow proper respiratory hygiene protocols, have adequate vaccination status, and that the time, frequency, and number of people required to enter ape habitats are minimized as much as possible [64].

We observed different patterns of infection among the four study sites. At Bwindi and Kibale, the prevalence of respiratory pathogens was higher than at Budongo and Bulindi. These differences could be due to stochastic effects resulting from the single cross-sectional nature of our study, such as attributes of the particular individuals who volunteered to participate or unknown circumstances during sampling. Alternatively, it could be due to demographic differences affecting respiratory pathogen susceptibility (e.g., participant ages, health care access, vaccination rates, exposure to fine particulate matter pollution, and population densities) or environmental differences affecting pathogen viability and transmission (e.g., meteorological factors, such as relative humidity and temperature). To address the causes of the observed variation among sites would likely require following cohorts of participants over time, as we have done at Kibale in the past [29].

Children were generally more likely than adults to have respiratory pathogens detected in their nasal swabs. This is consistent with results from our previous research at Kibale [29] and from around the world [41]. Children have developing adaptive immune systems [65], which increases both susceptibility to infection [7, 56] and duration of pathogen shedding [66]. Furthermore, children are frequently exposed to respiratory pathogens due to high rates of contact with other infected children in schools and daycares [67, 68]. Young children also tend to have higher viral titers than older children and adults, which increases the probability of transmission among children in this age group [69, 70]. In this light, we were surprised by the results from Bwindi showing the opposite trend, in which children had lower rates of pathogen detection than adults. Again, this result may have been due to the timing of sampling or to stochastic factors, but demographic or climatic factors (see above) at Bwindi might also have influenced the observed pattern.

Respiratory pathogens such as RV (overall prevalence: 50.4%), MPV (8.0%), PIV 3 (7.0%), CoV OC43 (5.2%), and RSV (3.0%) have been identified as the causative agents of respiratory disease outbreaks in populations of great apes near the study sites [15, 18] or elsewhere in sub-Saharan Africa [11, 14, 21, 71]. While frequencies of RV and RSV were similar to those in our previous longitudinal study at Kibale (57.9% and 4.0%, respectively), frequencies of MPV, PIV 3, and CoV OC43 were higher in the current study of sites across Uganda (3.1%, 0.7%, and 0.4%, respectively) [29]. Together, these viruses of known risk to great apes comprised nearly three quarters (73.6%) of total respiratory pathogen detections in these human communities near wild ape habitats. This observation indirectly supports our hypothesis that the source of these infections for apes may be the local human populations living near to, and interacting with, those apes. It remains unknown why these five viruses, and not other common human respiratory pathogens, account for diagnosed outbreaks in apes. Increased transmissibility to, or virulence in, apes of these particular viruses may account for their apparently disproportionate risk of causing high-consequence disease in wild apes.

We identified SARS-CoV-2 in three of the four locations we examined (we did not detect it in Bwindi). Again, stochastic factors may account for the absence of SARS-CoV-2 detections in Bwindi. Alternatively, the absence of SARS-CoV-2 detections in Bwindi might be attributed to the proactive measures taken by authorities and tourism operators in the vicinity of Bwindi National Park in response to the emergence of the COVID-19 pandemic [72]. These measures included the implementation of health and safety protocols to safeguard both visitors and local communities. Additionally, Bwindi National Park's location in a remote area with limited infrastructure and transportation options may serve as a natural barrier, preventing traffic of a large number of visitors from highly affected regions and thereby minimizing the risk of transmission.

SARS-CoV-2 and seasonal alphacoronaviruses were identified with relatively high frequencies (12.8% and 19.5% total prevalence, respectively) and were the most common respiratory pathogens identified at Bulindi, where chimpanzees inhabit a village environment and have particularly close, frequent interactions with local communities [38]. It is therefore interesting that no SARS-CoV-2 outbreaks have yet been documented in Uganda's apes. This could be because of strict biosecurity protocols that were implemented at the protected great ape sites in our study (Budongo, Bwindi, and Kibale) in response to COVID-19 [73], the lower risk of airborne transmission in outdoor environments than in crowded indoor settings [74], or other as-yet unidentified reasons. It is also possible that the absence of screening in most ape populations, particularly high-risk populations living outside of protected areas, including the Bulindi chimpanzees, could underestimate the effects of COVID-19 on wild apes. COVID-19 outbreaks have threatened zoo-housed gorillas in both the United States [75] and Europe [76, 77]. Indoor enclosures as well as close contact with zoo personnel and visitors may contribute to the increased transmission risk observed in captivity, but these outbreaks serve as proof that captive apes' wild conspecifics are also at high risk of infection and morbidity. In the current study, we detected a higher prevalence of SARS-CoV-2 than in our earlier study at Kibale (12.8% vs., 1.2%, respectively) [29], likely because the earlier study was conducted primarily during Uganda's COVID-19 lockdown period and the later study was conducted shortly after the Omicron variant wave. Continued vigilance and adherence to biosecurity guidelines regarding great ape interactions and COVID-19 [73] remain essential for protecting these animals despite loosening of the public health restrictions in place during 2020–2021. Ultimately, the goal is to support great ape conservation, but the proximal benefit can and should include greater emphasis on improved health outcomes for people working near these animals [27].

Our findings have implications for both human public health and great ape conservation. If, as our data suggest, the respiratory pathogens that most commonly threaten wild apes circulate at high frequencies in local human populations, implementing public health programs to reduce transmission within local human communities should have the indirect effect of reducing transmission risk to apes. Our previous findings from a cohort study in Kibale [29] showed the likeliest transmission pathway from humans to apes being through asymptomatically infected adults who became infected from children and unwittingly carried the pathogens into the forest to infect apes. Overall, our current data support this hypothesis, despite variation among sites likely due to unknown temporal, environmental, or stochastic factors. Furthermore, this study's inclusion of Bulindi, where wild chimpanzees range and forage in villages daily, interacting with human objects, structures, leftover food, and waste, is a reminder that additional complex transmission pathways have yet to be elucidated [79]. Our findings therefore support the value of a “One Health” approach to the problem. Indeed, we have initiated a program modeled on this approach that we have named “Healthy Children, Healthy Apes.” This program aims to reduce respiratory pathogen transmission in local primary school children through infrastructural and educational improvements to schools, with the expected benefit of reducing reverse zoonotic respiratory disease in nearby apes [78]. We hope that the ultimate solution to the real and growing problem of reverse zoonotic transmission of respiratory pathogens to wild apes may indeed lie in public health interventions directed toward the most vulnerable people in populations sharing habitats with apes.

## Figures and Tables

**Table 1:** Demographic and data collection information for each of the four study sites.

Study site	Month of sample collection	Adults (number of swabs)	Children (number of swabs)	Total (number of swabs)
Budongo	June 2022	25 (25)	79 (79)	104 (104)
Bulindi	June 2022	28 (28)	75 (75)	103 (103)
Bwindi	October 2022	55 (55)	45 (45)	100 (100)
Kibale	February and July 2022	19 (19)	70 (106)	89 (125)
Total	—	127 (127)	269 (305)	396 (432)

**Table 2:** Frequencies of respiratory symptoms and respiratory pathogen infections for the three sites reporting symptoms (Budongo, Bulindi, and Kibale).

Cohort	S+/P+ (%)	S−/P+ (%)	S+/P− (%)	S−/P− (%)
Budongo	26	1	66	13
Adults	2	0	17	6
Children	24	1	49	7
Bulindi	20	2	74	6
Adults	6	0	20	3
Children	14	2	54	3
Kibale	28	8	51	27
Adults	0	1	6	12
Children	28	7	45	15
Total adults	8 (11.0)	1 (1.4)	43 (58.9)	21 (28.8)
Total children	66 (26.5)	10 (4.0)	148 (59.4)	25 (10.0)
Total participants	74 (23.0)	11 (3.4)	191 (59.3)	46 (14.3)

Abbreviations: S+/P+, symptoms positive/pathogen positive (symptomatic infection); S−/P+, symptoms negative/pathogen positive (asymptomatic infection); S+/P−, symptoms positive/pathogen negative (nonviral cold); and S−/P−, symptoms negative/pathogen negative (healthy).

## Data Availability

The data that support the findings of this study are available from the corresponding author (Tony Goldberg) upon reasonable request.

## Supporting Information

Supplementary Materials Tables S1–S4 list prevalence rates of pediatric respiratory pathogens detected in nasopharyngeal swabs in people living near Budongo Central Forest Reserve, Bulindi Town Council, Bwindi Impenetrable National Park, and Kibale National Park, respectively.
