# Supplementary material for: Pediatric Respiratory Pathogens Circulate in Children and Adults in Communities Near Susceptible Wild Great Ape Populations in Uganda
Source: Transbound Emerg Dis. 2024 Oct 23;2024:1771163. doi: 10.1155/2024/1771163 (PMC12016897; doi:10.1155/2024/1771163)
Supplement: Supplementary Materials — Tables S1–S4 list prevalence rates of pediatric respiratory pathogens detected in nasopharyngeal swabs in people living near Budongo Central Forest Reserve, Bulindi Town Council, Bwindi Impenetrable National Park, and Kibale National Park, respectively. [file 1771163.f1.docx]

**Supplementary Materials**

**Table S1:** Respiratory pathogens detected in nasopharyngeal swabs collected from people living near Budongo Central Forest Reserve, June 2022 (n=25 out of 104 swabs, 24.0%).

| **Pathogen** | **Adult Cases**  n (%) | **Child Cases**  n (%) | **Total**  n (%) |
| --- | --- | --- | --- |
| Adenovirus | 0 (0.0) | 0 (0.0) | 0 (0.0) |
| Bocavirus | 0 (0.0) | 1 (4.0) | 1 (4.0) |
| Coronavirus (CoV) | 0 (0.0) | 8 (32.0) | 8 (32.0) |
| CoV 229E | 0 (0.0) | 0 (0.0) | 0 (0.0) |
| CoV HKU1 | 0 (0.0) | 1 (4.0) | 1 (4.0) |
| CoV NL63 | 0 (0.0) | 1 (4.0) | 1 (4.0) |
| CoV OC43 | 0 (0.0) | 0 (0.0) | 0 (0.0) |
| SARS-CoV-2 | 0 (0.0) | 6 (24.0) | 6 (24.0) |
| Influenza virus | 1 (4.0) | 2 (8.0) | 2 (8.0) |
| Influenza A (2009 H1N1) | 0 (0.0) | 0 (0.0) | 0 (0.0) |
| Influenza B | 1 (4.0) | 2 (8.0) | 2 (8.0) |
| Metapneumovirus (MPV) | 0 (0.0) | 1 (4.0) | 1 (4.0) |
| Parainfluenza virus (PIV) | 0 (0.0) | 0 (0.0) | 0 (0.0) |
| PIV 1 | 0 (0.0) | 0 (0.0) | 0 (0.0) |
| PIV 2 | 0 (0.0) | 0 (0.0) | 0 (0.0) |
| PIV 3 | 0 (0.0) | 0 (0.0) | 0 (0.0) |
| PIV 4 | 0 (0.0) | 0 (0.0) | 0 (0.0) |
| Respiratory syncytial virus (RSV) | 1 (4.0) | 0 (0.0) | 1 (4.0) |
| RSV A | 1 (4.0) | 0 (0.0) | 1 (4.0) |
| RSV B | 0 (0.0) | 0 (0.0) | 0 (0.0) |
| Rhinovirus/enterovirus (RV) | 0 (0.0) | 11 (44.0) | 11 (44.0) |
| *Chlamydophila pneumoniae* | 0 (0.0) | 0 (0.0) | 0 (0.0) |
| *Legionella pneumophila* | 0 (0.0) | 0 (0.0) | 0 (0.0) |
| *Mycoplasma pneumoniae* | 1 (4.0) | 1 (4.0) | 2 (8.0) |
| **Total** | **3** | **22** | **25** |

**Table S2:** Respiratory pathogens detected in nasopharyngeal swabs collected from people living near the Bulindi Chimpanzee and Community Project, June 2022 (n=21 out of 101 swabs, 20.8%). Total positive cases are greater than total positive swabs due to presence of coinfections.

| **Pathogen** | **Adult Cases**  n (%) | **Child Cases**  n (%) | **Total**  n (%) |
| --- | --- | --- | --- |
| Adenovirus | 0 (0.0) | 0 (0.0) | 0 (0.0) |
| Bocavirus | 0 (0.0) | 1 (4.3) | 1 (4.3) |
| Coronavirus (CoV) | 3 (13.0) | 11 (47.8) | 14 (60.9) |
| CoV 229E | 0 (0.0) | 0 (0.0) | 0 (0.0) |
| CoV HKU1 | 0 (0.0) | 0 (0.0) | 0 (0.0) |
| CoV NL63 | 0 (0.0) | 5 (17.4) | 5 (17.4) |
| CoV OC43 | 0 (0.0) | 3 (13.0) | 3 (13.0) |
| SARS-CoV-2 | 3 (13.0) | 3 (13.0) | 6 (26.1) |
| Influenza virus | 0 (0.0) | 0 (0.0) | 0 (0.0) |
| Influenza A (2009 H1N1) | 0 (0.0) | 0 (0.0) | 0 (0.0) |
| Influenza B | 0 (0.0) | 0 (0.0) | 0 (0.0) |
| Metapneumovirus (MPV) | 0 (0.0) | 1 (4.3) | 1 (4.3) |
| Parainfluenza virus (PIV) | 0 (0.0) | 0 (0.0) | 0 (0.0) |
| PIV 1 | 0 (0.0) | 0 (0.0) | 0 (0.0) |
| PIV 2 | 0 (0.0) | 0 (0.0) | 0 (0.0) |
| PIV 3 | 0 (0.0) | 0 (0.0) | 0 (0.0) |
| PIV 4 | 0 (0.0) | 0 (0.0) | 0 (0.0) |
| Respiratory syncytial virus (RSV) | 0 (0.0) | 0 (0.0) | 0 (0.0) |
| RSV A | 0 (0.0) | 0 (0.0) | 0 (0.0) |
| RSV B | 0 (0.0) | 0 (0.0) | 0 (0.0) |
| Rhinovirus/enterovirus (RV) | 2 (8.7) | 5 (17.4) | 7 (30.4) |
| *Chlamydophila pneumoniae* | 0 (0.0) | 0 (0.0) | 0 (0.0) |
| *Legionella pneumophila* | 0 (0.0) | 0 (0.0) | 0 (0.0) |
| *Mycoplasma pneumoniae* | 0 (0.0) | 0 (0.0) | 0 (0.0) |
| **Total** | **5** | **18** | **23** |

**Table S3:** Respiratory pathogens detected in nasopharyngeal swabs collected from people living near Bwindi Impenetrable National Park, October 2022 (n=44 out of 100 swabs, 44.0%). Total positive cases are greater than total positive swabs due to presence of coinfections.

| **Pathogen** | **Adult Cases**  n (%) | **Child Cases**  n (%) | **Total**  n (%) |
| --- | --- | --- | --- |
| Adenovirus | 8 (15.7) | 0 (0.0) | 8 (15.7) |
| Bocavirus | 0 (0.0) | 0 (0.0) | 0 (0.0) |
| Coronavirus (CoV) | 4 (7.8) | 1 (2.0) | 5 (9.8) |
| CoV 229E | 0 (0.0) | 0 (0.0) | 0 (0.0) |
| CoV HKU1 | 4 (7.8) | 0 (0.0) | 4 (7.8) |
| CoV NL63 | 0 (0.0) | 1 (2.0) | 1 (2.0) |
| CoV OC43 | 0 (0.0) | 0 (0.0) | 0 (0.0) |
| SARS-CoV-2 | 0 (0.0) | 0 (0.0) | 0 (0.0) |
| Influenza virus | 0 (0.0) | 0 (0.0) | 0 (0.0) |
| Influenza A (2009 H1N1) | 0 (0.0) | 0 (0.0) | 0 (0.0) |
| Influenza B | 0 (0.0) | 0 (0.0) | 0 (0.0) |
| Metapneumovirus (MPV) | 5 (9.8) | 0 (0.0) | 5 (9.8) |
| Parainfluenza virus (PIV) | 2 (3.9) | 7 (13.7) | 9 (17.6) |
| PIV 1 | 0 (0.0) | 0 (0.0) | 0 (0.0) |
| PIV 2 | 0 (0.0) | 0 (0.0) | 0 (0.0) |
| PIV 3 | 1 (2.0) | 7 (13.7) | 8 (15.7) |
| PIV 4 | 1 (2.0) | 0 (0.0) | 1 (2.0) |
| Respiratory syncytial virus (RSV) | 3 (5.9) | 0 (0.0) | 3 (5.9) |
| RSV A | 3 (5.9) | 0 (0.0) | 3 (5.9) |
| RSV B | 0 (0.0) | 0 (0.0) | 0 (0.0) |
| Rhinovirus/enterovirus (RV) | 12 (23.5) | 9 (17.6) | 21 (41.2) |
| *Chlamydophila pneumoniae* | 0 (0.0) | 0 (0.0) | 0 (0.0) |
| *Legionella pneumophila* | 0 (0.0) | 0 (0.0) | 0 (0.0) |
| *Mycoplasma pneumoniae* | 0 (0.0) | 0 (0.0) | 0 (0.0) |
| **Total** | **34** | **17** | **51** |

**Table S4:** Respiratory pathogens detected in nasopharyngeal swabs collected from people living near Kibale National Park, February and July, 2022 (n=43 out of 125 swabs, 34.4%). Total positive cases are greater than total positive swabs due to presence of coinfections.

| **Pathogen** | **Adult Cases**  n (%) | **Child Cases**  n (%) | **Total**  n (%) |
| --- | --- | --- | --- |
| Adenovirus | 0 (0.0) | 1 (2.0) | 1 (2.0) |
| Bocavirus | 0 (0.0) | 2 (4.0) | 2 (4.0) |
| Coronavirus (CoV) | 0 (0.0) | 13 (26.0) | 13 (26.0) |
| CoV 229E | 0 (0.0) | 0 (0.0) | 0 (0.0) |
| CoV HKU1 | 0 (0.0) | 2 (4.0) | 2 (4.0) |
| CoV NL63 | 0 (0.0) | 4 (8.0) | 4 (8.0) |
| CoV OC43 | 0 (0.0) | 4 (8.0) | 4 (8.0) |
| SARS-CoV-2 | 0 (0.0) | 3 (6.0) | 3 (6.0) |
| Influenza virus | 0 (0.0) | 2 (4.0) | 2 (4.0) |
| Influenza A (2009 H1N1) | 0 (0.0) | 1 (2.0) | 1 (2.0) |
| Influenza B | 0 (0.0) | 1 (2.0) | 1 (2.0) |
| Metapneumovirus (MPV) | 0 (0.0) | 3 (6.0) | 3 (6.0) |
| Parainfluenza virus (PIV) | 0 (0.0) | 2 (4.0) | 2 (4.0) |
| PIV 1 | 0 (0.0) | 1 (2.0) | 1 (2.0) |
| PIV 2 | 0 (0.0) | 0 (0.0) | 0 (0.0) |
| PIV 3 | 0 (0.0) | 1 (2.0) | 1 (2.0) |
| PIV 4 | 0 (0.0) | 0 (0.0) | 0 (0.0) |
| Respiratory syncytial virus (RSV) | 0 (0.0) | 0 (0.0) | 0 (0.0) |
| RSV A | 0 (0.0) | 0 (0.0) | 0 (0.0) |
| RSV B | 0 (0.0) | 0 (0.0) | 0 (0.0) |
| Rhinovirus/enterovirus (RV) | 1 (2.0) | 26 (52.0) | 27 (52.0) |
| *Chlamydophila pneumoniae* | 0 (0.0) | 0 (0.0) | 0 (0.0) |
| *Legionella pneumophila* | 0 (0.0) | 0 (0.0) | 0 (0.0) |
| *Mycoplasma pneumoniae* | 0 (0.0) | 0 (0.0) | 0 (0.0) |
| **Total** | **1** | **49** | **50** |
